# An Institutional Febrile Neutropenia Protocol Improved the Antibacterial Treatment and Encouraged the Development of a Computerized Clinical Decision Support System

**DOI:** 10.3390/antibiotics13090832

**Published:** 2024-09-02

**Authors:** Zahit Taş, Gökhan Metan, Gülçin Telli Dizman, Eren Yavuz, Ömer Dizdar, Yahya Büyükaşık, Ömrüm Uzun, Murat Akova

**Affiliations:** 1 Department of Infectious Diseases and Clinical Microbiology, Hacettepe University Faculty of Medicine, Ankara 06100, Turkey; gokhanmetan@hacettepe.edu.tr (G.M.); gulcin.telli@hacettepe.edu.tr (G.T.D.); makova@hacettepe.edu.tr (M.A.); 2 Hemosoft Software Development Department, Ankara 06800, Turkey; 3 Department of Medical Oncology, Hacettepe University Faculty of Medicine, Ankara 06230, Turkey; 4 Department of Hematology, Hacettepe University Faculty of Medicine, Ankara 06100, Turkey

**Keywords:** cancer, hematological malignancy, solid tumors, febrile neutropenia, febrile neutropenia guidelines, hematopoietic stem cell transplantation, computerized clinical decision support system

## Abstract

We investigated the influence of a local guideline on the quality of febrile neutropenia (FN) management and the applicability of a computerized decision support system (CDSS) using real-life data. The study included 227 FN patients between April 2016 and January 2019. The primary outcome measure was the achievement of a 20% increase in the rate of appropriate empirical treatment of FN in bacteremic patients. The compatibility of the CDSS (the development of which was completed in November 2021) with local protocols was tested using standard patient scenarios and empirical antibiotic recommendations for bacteremic FN patients. In total, 91 patients were evaluated before (P1: between April 2016 and May 2017) and 136 after (P2: between May 2017 and January 2019) the guideline’s release (May 2017). The demographic characteristics were similar. Appropriate empirical antibacterial treatment was achieved in 58.3% of P1 and 88.1% of P2 patients (*p* = 0.006). The need for escalation of antibacterial treatment was significantly lower in P2 (49.5% vs. 35.3%; *p* = 0.03). In P2, the performance of the CDSS and consulting physicians was similar (CDSS 88.8% vs. physician 88.83%; *p* = 1) regarding appropriate empirical antibacterial treatment. The introduction of the local guideline improved the appropriateness of initial empirical treatment and reduced escalation rates in FN patients. The high rate of compliance of the CDSS with the local guideline-based decisions in P2 highlights the usefulness of the CDSS for these patients.

## 1. Introduction

Febrile neutropenia (FN) is a potentially life-threatening complication in patients receiving myelosuppressive treatment. The implementation of effective diagnostic and antimicrobial stewardship strategies is a crucial aspect of the successful management of FN [1]. Several FN guidelines have been prepared by various international societies and organizations. The National Comprehensive Cancer Network (NCCN) and Infectious Diseases Society of America (IDSA) FN guidelines offer largely analogous recommendations during the initial assessment. The IDSA guideline, which assesses FN in both outpatient and hospitalized patients, is more comprehensive than European guidelines in the initial assessment [2,3,4]. The American Society for Clinical Oncology (ASCO), NCCN, and IDSA recommend fluoroquinolone prophylaxis for high-risk patients, specifically those expected to have profound and prolonged neutropenia, whereas the European and Australian guidelines are more rigorous in their recommendations for antibiotic prophylaxis [4,5,6]. This is because no advantage in terms of mortality with such prophylaxis has been proven; there is also an increased risk of adverse effects, and the risk of multidrug resistant bacterial infections is a significant concern. Recently, the 2021 updated European Conference on Infections in Leukemia (ECIL) guidelines reviewed the risk of infections and FN associated with cytotoxic treatments; their recommendation for antibacterial prophylaxis is limited to instances where hypomethylating agents are combined with venetoclax [7]. Furthermore, there are discrepancies regarding the optimal duration of broad-spectrum antibacterial therapy in patients with FN. In accordance with the guidance provided by the IDSA, the standard practice has been to continue with broad-spectrum antibiotic treatment until the patient has recovered from neutropenia. The NCCN proposes de-escalation or discontinuation in certain circumstances. However, the ECIL guidelines advise that the initial regimen should be modified at 72 to 96 h based on the patient’s clinical course and microbiological results. The discontinuation of antibiotics, irrespective of the neutrophil count, should be considered with an expected duration of neutropenia of more than 72 h in neutropenic patients with a fever of unknown origin who are hemodynamically stable and afebrile for 48 h; later, it may be considered irrespective of the neutrophil count or the expected duration of neutropenia. A comparative analysis of the recommendations of the FN guidelines in different clinical situations has been the subject of a comprehensive review [1].

It has been demonstrated that increased compliance with these guidelines is beneficial in numerous areas, including a reduction in the incidence of febrile neutropenic patients requiring transfer to intensive care and the decreased consumption of antibiotics such as glycopeptides, meropenem, and colistin [8,9,10]. The availability of management guidelines was found to be associated with a reduced mortality rate in FN [11]. Although these guidelines are evidence-based and readily available online, the potential for language barriers hinders their effective implementation in a busy on-call setting, particularly for junior physicians unfamiliar with their use [2,3,4]. An international survey that covered 567 centers in Asia and Europe showed that the recommendations put forward in the European guidelines are not widely implemented in clinical practice [12]. The adaptation of international guidelines into local settings through the establishment of institutional guidelines can be beneficial when taking into account the availability of diagnostic tests, the discrepancy in the epidemiology of antimicrobial resistance, and the accessibility of antimicrobial agents. In this context, the establishment of institutional antimicrobial stewardship programs is of paramount importance. The implementation of antimicrobial stewardship programs has been demonstrated to have a beneficial impact on a range of outcomes, including durations of hospital stay, readmission rates, and mortality [13]. The use of artificial intelligence has seen more attention in antimicrobial treatment, and there are studies indicating the use of artificial intelligence in the management of FN [14]. A computerized clinical decision support system (CDSS) might represent a pivotal element in the effective development of artificial intelligence.

The first electronic health record (EHR) was in use almost six decades ago. Such systems may employ a range of functionalities, from simple pop-up alerts about serious drug allergies to more sophisticated tools incorporating clinical prediction rules [15]. A CDSS can be defined as computer software that utilizes the characteristics of a particular individual patient in order to present patient-specific assessments or recommendations to the clinician in order to facilitate a decision.

CDSSs are tools that have been considered important candidates to improve antimicrobial stewardship in different settings. A review of the literature reveals that between 1975 and 2017, more than 2000 studies demonstrated the efficacy of CDSSs in enhancing the quality of healthcare across a range of medical specialties [16]. Over the past two decades, there has been growing attention given to the role of CDSSs in antimicrobial stewardship [17]. A meta-analysis of 46 studies on the role of CDSSs in antimicrobial stewardship found a statistically significant benefit of CDSS use on ≥1 study variable in 42 of the 46 studies. The use of CDSSs resulted in a statistically significant increase in the percentage accuracy of the empirically prescribed antibiotic spectrum with respect to microbial susceptibility in six out of seven studies, all from different hospital settings. In addition, 10 studies reported an increase in adherence to prescribing guidelines when CDSSs were used [18]. Although CDSSs have been used in various areas of antimicrobial stewardship, there has been limited information on the use of CDSSs in febrile neutropenic patients [19].

In our hospital, antifungal, antibacterial, and antiviral prophylaxis is initiated by hematology or oncology consultants for cancer patients or recipients of hematopoietic stem cell transplantation (HSCT). However, the decisions about the treatment of FN are made by infectious disease (ID) specialists. Requests for screening and diagnostic tests are made by internal medicine residents who are in the first two years of their residency program following consultants in hematology/oncology and infectious diseases. Senior consultants are familiar with the IDSA, NCCN, and ECIL guidelines, but there was no official institutional guideline in place until May 2017.

In this study, we investigated the impact of a guideline in the local language (Turkish) on the quality of the management of FN in a setting with a complex hierarchy regarding the prevention, diagnosis, and treatment of FN. We also tested the applicability of a CDSS in the management of FN patients with bacteremia.

## 2. Results

### 2.1. Patient Baseline Characteristics

A total of 227 patients at least 18 years of age who were diagnosed with FN between 8 April 2016 and 7 January 2019 were included. The median age of the patients was 53 (minimum 18; maximum 82) years, and 143 (78.1%) were male. In total, 175 (77.1%) patients had a hematological malignancy, 26 had undergone allogeneic HSCT, and 40 had undergone autologous HSCT. A total of 91 (40.1%) out of the 227 patients were treated for FN before the release of the local guideline (Period 1: P1), and the remaining 136 (59.9%) patients were treated after the release of the local guideline (second period: P2). The demographic characteristics of the patients were similar except for the duration of neutropenia (Table 1).

### 2.2. Prophylaxis

Full compliance with the local guideline recommendations for antibacterial prophylaxis was detected in 11 (33.3%) out of 33 patients in P1 and in 25 of 61 patients (40.9%) in P2 (*p* = 0.53). Full compliance for antifungal prophylaxis was twice as high in P1 (21.2% compared to 10.8 in P2; *p* = 0.19). The most common reason for noncompliance with antibacterial and antifungal prophylaxis was the timing of initiation and cessation of prophylaxis (Table 2).

### 2.3. Diagnostic Stewardship

Blood cultures were drawn in 223 (98.2%) of 227 patients with FN. It is worth noting that only two patients (2/91; 2.2%) had two sets of blood cultures in P1, while there was a notable increase in the number of patients with two sets of blood cultures in P2 (34/136, 25.0%) (*p* < 0.001). Galactomannan (GM) antigen screening was indicated in 84 patients. The compliance with the local protocol regarding GM screening was 80% (28 of 35 patients) in P1 and 75.5% (37 of 49 patients) in P2 (*p* = 0.64). The rate of compliance with CMV PCR for screening purposes was 89.5% (17 of 19 patients) in P1 and 76% (19 of 25 patients) in P2 (*p* = 0.35). Diarrhea developed in 68 (30%) of the patients. *Clostridioides difficile* toxin B PCR was requested in 24 (88.9%) of 27 patients with diarrhea in P1 and in 37 (90.2%) of 41 patients with diarrhea in P2 (*p* = 1.0).

### 2.4. Antibacterial Stewardship

Blood cultures were positive in 76 (33.6%) patients. In 66 episodes (29.1%), the pathogen was accepted as the causative agent. The most common bacteria isolated from blood cultures were *Escherichia coli* in 41 patients, followed by *Klebsiella pneumoniae* in 7, *Pseudomonas aeruginosa* in 4, and *Serratia marcescens*, *Acinetobacter baumannii*, and *Bacteroides fragilis* in 1 patient each. Coagulase-negative staphylococci were the most common Gram-positive bacteria, but only 46.1% of 13 Gram-positive bacteria were accepted as the etiologic agent of bloodstream infections. Methicillin-susceptible *Staphylococcus aureus* was isolated in the blood cultures of two patients.

There was no statistically significant difference between the two periods regarding risk factors for antibacterial resistance, indications for empirical glycopeptide treatment, and antibacterial resistance rates (Table 3). According to the antibacterial susceptibility test, the empirical antibacterial treatment was appropriate in 51 (77.3%) of the patients with a positive blood culture. The rate of appropriate empirical antibacterial therapy in bacteremic patients in P1 (58.3%) increased significantly in P2 (88.1%; *p* = 0.006) (Table 4).

Fever resolved with the initial empirical antibacterial treatment in 35.2% of the patients in P1 and 58.1% in P2 (*p* = 0.001). Overall, the initial empirical antibacterial treatment was escalated in 93 (41%) out of the 227 patients. The need for escalation was significantly lower in P2 (P1; 49.5% vs. P2; 35.3%; *p* = 0.03), and it was carried out later in P2 (median 3 days in P1 vs. median 4 days in P2; *p* = 0.03). The most common reason for escalation was persistent fever under empirical treatment, followed by clinical deterioration and bloodstream infection by a resistant bacterium (Table 4). More patients received antibacterial treatment for ≤7 days in P2 compared to P1 (34.5% vs. 21.9%; *p* = 0.02). The 30-day crude mortality rate was similar in both periods (*p* = 0.38) (Table 4).

### 2.5. Compliance with the CDSS

Four residents and three infectious disease specialists evaluated a series of 50 scenarios that represent common occurrences in clinical practice regarding the CDSS. The recommendations provided by the CDSS were found to be 100% in line with the established local guideline. In the second part, the clinical descriptive characteristics of 66 bacteremic patients were entered into the CDDS. The recommended antibiotic was appropriate according to the antibiogram result in 80.3% (53 of 66) of the CDSS recommendations. The CDSS did slightly better than the consulting physician in P1 (CDSS 66.3% vs. physician 58.3%; *p* = 0.5) in regard to the appropriateness of the initial empirical antibacterial treatment in this subset of patients. The performance of the CDSS and the consulting physician was similar in P2 (CDSS 88.8% vs. physician 88.83%, *p* = 1) (Appendix A).

## 3. Discussion

In this study, we observed that the quality of antibacterial treatment for FN was improved after the introduction of a guideline provided in the local language (Turkish). The situation in our study was somehow different from the situations in previously published studies. The ID team made decisions about antibacterial treatments in each period by using the same international guidelines. The difference between the two periods was the availability of a written and officially released local protocol in the local language, which was easily accessible from the hospital information system. A written protocol with continuous discussions on compliance with the local guideline during rounds might lead to greater interest in and use of the algorithm. A recent study from our center showed that continuous audits and reporting increased compliance with local treatment protocols not only for FN but also for all local antibacterial treatment algorithms [21]. Despite there being no significant change in the epidemiology of bloodstream infections and the risk factors for antibacterial resistance between two periods, the rate of fever resolution with the initial empirical antibiotics was higher in P2 (Table 3). In addition, the rate of appropriateness of antibacterial treatment in patients with bloodstream infections was higher in P2, and the rate of escalation in this period was lower (Table 4). Although the patient care teams tried to follow the same international guidelines in both periods, an emphasis on the local protocol during rounds and educational meetings could possibly lead to an improvement in the selection of empirical treatment when experienced ID consultants are only on-call, such as at night or on weekend shifts.

The patients included in the analysis were randomly selected by the infection control committee during the specified periods to assess adherence to FN protocols. Despite the lack of randomization, the demographic characteristics were similar between P1 and P2, except for the neutropenia duration. This suggests that similar groups were assessed for adherence to the guidelines both before and after its implementation. A longer duration of antibiotic therapy might have been expected in the second period, where neutropenia lasted longer. Although the duration of neutropenia was longer in P2, more patients received empirical antibacterial treatment for less than 7 days in P2 compared to P1. This could be the result of increased awareness of the guideline to discontinue antibiotics in clinically stable neutropenic patients without any proven or suspected bacterial infection (Table 4). A recent systematic review of 11 randomized controlled studies that included 1128 distinct patients with FN did not find any difference between short-term antibacterial treatment and long-term antibacterial treatment in terms of mortality, clinical failure, and relapsing bacteremia [22].

Although the majority of FN guidelines recommend antifungal prophylaxis to prevent invasive fungal infections, a previous study including 549 high-risk hematology patients and recipients of HSCT reported a high rate of deviation from the integrated institutional pathway [23]. An electronic survey from Spain showed poor compliance rates among transplant physicians regarding antifungal prophylaxis [24]. In this study, the appropriate use of antifungal prophylaxis was high in both periods (91.6% in P1 and 97.3% in P2). While the compliance rates with the type and dosages of antifungal drugs were sufficient, compliance with the timing of initiation and cessation was poor. These findings were similar for antibacterial prophylaxis. Compliance with the timing of initiation and cessation was poor as 33% in P1 and 41% in P2. This problem may be fixed by integrating the CDSS into the hospital information system.

Although CDSSs have been used in various areas of antimicrobial stewardship, there is limited information on the use of CDSSs in febrile neutropenic patients [19]. In our study, the performance of the CDSS and consulting physician was similar in P2, with rates of 88.8% regarding selection of the appropriate empirical antibacterial treatment. In a cluster-randomized cross-over study, a prospective review and feedback regarding antibiotic prescriptions or compulsory CDSSs did not result in different clinical outcomes, antibiotic durations, or lengths of stay [25]. A CDSS may be more useful in a setting where decisions regarding antibiotics are made by a junior physician or in the absence of a structured management protocol. Some concerns have been raised about CDSSs, such as a potential risk of reducing critical thinking and professional autonomy among physicians, in addition to new medico-legal issues [26]. Because our CDSS runs with a decision-tree algorithm, physician inputs are critical. This factor has the potential to allow the physician to remain actively involved in the decision-making process.

### Study Limitations

Our study was conducted at a single center and included only patients hospitalized with FN in the oncology hospital during a specific period. It is possible that some confounding variables may have been underestimated due to the retrospective design. Since the study was retrospective, complete data for some patients were not accessible, and there is a possibility of inaccurate records. The number of patients included in the evaluation was affected by missing data in the assessment of adherence to antibiotic and antifungal prophylaxis as well as by the fact that some patients were already under treatment when they developed FN, which may have influenced the adherence results. It is not possible to ascertain compliance with the local protocol with 100% accuracy since we were unable to include all patients with FN in the study period. The primary objective of the protocol was to enhance the quality of care when an ID consultant could not be present at the patient’s bedside. However, due to the limitations of the study, it was not feasible to categorize the treatment selections made at the bedside or during on-call shifts during night and weekend periods. We did not have sufficient data on antibiotic side effects across the different periods, which is one of this study’s limitations. Local pathogen profiles and resistance profiles can vary between different hospitals and wards. This may influence the appropriateness of empirical treatment. Since this was a study conducted at a single center, the results cannot be extrapolated to other centers and settings. Additionally, blood culture volumes and the number of blood culture sets obtained are directly associated with the detection of bacteremia. The CDSS recommends performing two sets of blood cultures in accordance with the local guidelines, which were developed based on international guideline recommendations. Although it was recommended to obtain two sets of blood cultures in patients with FN, in most cases, only one set of blood cultures was obtained. Therefore, optimal blood culture efficacy might not have been achieved.

## 4. Material and Methods

### 4.1. Establishment of the Local Guideline

This study was conducted in the Hacettepe University Faculty of Medicine Oncology Hospital, which has 119 beds, including an 8-bed intensive care unit and a 16-bed hematopoietic stem cell transplantation (HSCT) ward. In our hospital, febrile neutropenic patients are attended by an experienced infectious disease (ID) consultant with a team of junior doctors (either ID trainees or internal medicine residents working in the ID department whose primary responsibilities are to assess and prepare the patient for ID consultation, then follow up on the patient’s clinical condition and laboratory test results and inform the ID consultant) during work hours, but those consulted during night shifts or on the weekends are evaluated initially by junior doctors (ID trainees and internal medicine residents working in the ID department evaluate the patients at bedside and then call an ID consultant for treatment decisions). Diagnostic tests and screening tests are usually requested beforehand by the junior doctors in the first or second year of their internal medicine residency, who are on duty at night or during weekend shifts; this may lead to inappropriate test demands, such as galactomannan antigen from a patient under mold prophylaxis without suspicion of invasive aspergillosis, or CMV PCR in a patient with neutropenia. In order to overcome the potential risk of suboptimal diagnostic tests and treatment, an institutional guideline for the management of FN was released in May 2017.

The recommendations in international guidelines [2,3,4] were structured as the local guideline for the management of FN in collaboration with the hematology and oncology departments. Then, the guideline was distributed to all wards in the oncology hospital and included as an electronic document in the hospital information system, which was available to all physicians. This guideline was used as a basis for education and consultations. Lectures on the management of FN with an emphasis on the local guideline were given to all internal medicine and ID residents at least once a year.

### 4.2. Definitions, Inclusion Criteria, and Exclusion Criteria

**Figure 1:**
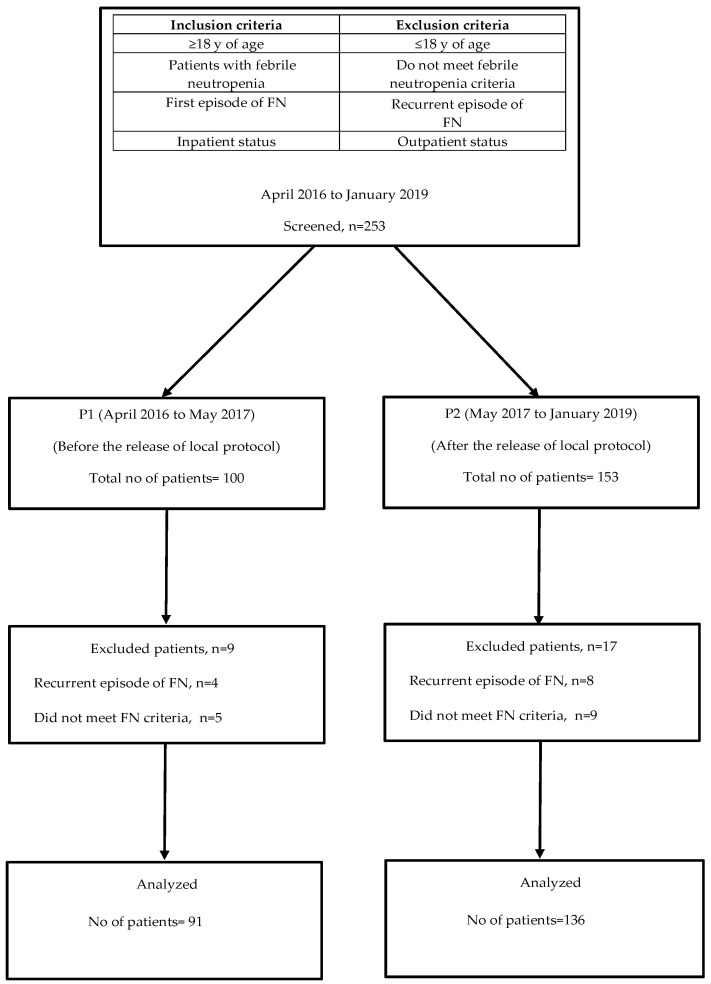
Study enrollment chart. FN (Febrile neutropenia).

FN was defined as a single tympanic temperature of ≥38.3 °C, or a temperature of ≥38 °C for at least 1 h with an absolute neutrophilic count (ANC) of less than 500 cells/mm^3^, or an ANC expected to decrease below 500 cells/mm^3^ in the next 48 h [3]. The patients included in the analysis were randomly selected by the infection control committee during the specified periods to assess adherence to FN protocols. A total of 253 patient records were reviewed, with 227 meeting the inclusion criteria. Among the remaining 26 patients, 12 had recurrent FN episodes, and 14 did not meet the FN criteria. These 26 patients were excluded from the study (Figure 1).

The demographic and clinical characteristics of the patients were reviewed by using Hacettepe Oncology Hospital Infection Control Committee Antimicrobial Stewardship Working Group Febrile Neutropenia Follow-up forms. All collected demographic, clinical, and microbiological data were checked against the hospital’s electronic medical record system. The ID consultation and follow-up notes, as well as discharge summaries, were used to evaluate the data, particularly for the antibacterial treatments and any microbiologically or clinically diagnosed infectious diseases. Compliance with the local guideline and the rate of appropriate empirical antibacterial treatment in patients with bacteremia were compared before and after the release of the guideline. Appropriate empirical therapy was defined as one or more agents active against the bacteria isolated in a blood culture, administered at an adequate dosage and via an appropriate route of administration, not later than 24 h after the blood culture was obtained. Compliance with the local guideline for antibacterial and antifungal prophylaxis was assessed for the indication, drug type, time of initiation, and discontinuation. Prophylaxis was defined as being fully compliant if it met all guideline recommendations. The study was approved by the Ethical Committee of Hacettepe University School of Medicine (approval number and date: GO 20/445; dated 22 May 2020). Data were used anonymously at all stages of the study. Informed consent was not required due to the retrospective nature of the study, in line with local regulations.

### 4.3. Guideline Recommendations

#### 4.3.1. Antibacterial and Antifungal Prophylaxis

Levofloxacin (500 mg/day, peroral (po)) was recommended as an antibacterial prophylaxis for patients with neutropenia expected to last longer than 7 days to start cytotoxic chemotherapy and stop after recovery from neutropenia (ANC > 500 cells/mm^3^ for 3 consecutive days) or the initiation of a systemic antibiotic for FN. Posaconazole delayed-release tablets (loading 600 mg/day, po, followed by maintenance 300 mg/day, po) were recommended as antifungal prophylaxis for patients who received induction chemotherapy for acute myeloid leukemia (AML), patients with myelodysplastic syndrome receiving AML-like chemotherapy, and recipients of allogeneic HSCT with grade 3 or 4 graft-versus-host disease (GVHD). Fluconazole (400 mg/day; po) was recommended as an antifungal prophylaxis for recipients of HSCT during the pre-engraftment phase and patients with neutropenia (<500 cells/mm^3^) expected to last longer than 7 days with a high risk of mucositis. If there was an interaction between azoles and a cytotoxic regimen, such as QT prolongation due to posaconazole and idarubicin co-administration, azole prophylaxis was started after the cancer drugs were completed. Otherwise, antifungal prophylaxis use commenced with cancer chemotherapy and stopped after recovery from neutropenia (ANC > 500 cells/mm^3^ for 7 consecutive days) or the initiation of antifungal therapy for the empirical treatment of persistent fever or invasive fungal disease.

#### 4.3.2. Diagnostic Stewardship

The local guideline recommends that two sets of blood cultures (to achieve the optimal volume and mitigate false positives triggered by contamination, drawn for each collection from two separate insertion sites) be taken from all patients before empirical antibacterial treatment is initiated. Galactomannan antigen screening was advised twice weekly in patients with neutropenia expected to last longer than 7 days if they did not receive mold-active prophylaxis. Thoracic computed tomography (CT) was requested in the case of a persistent fever for more than 72 h, a serum galactomannan antigen index of >0.5, regardless of fever, and/or any respiratory symptoms. Paranasal sinus CT was requested if there was a suspicion of sinusitis. *Clostridioides difficile* PCR was recommended in patients with diarrhea. Cytomegalovirus (CMV) PCR screening twice weekly was recommended in patients who received HSCT or high-dose chemotherapy for acute lymphocytic leukemia.

#### 4.3.3. Antibacterial Stewardship

Piperacillin–tazobactam, cefepime, and ceftazidime, either as a monotherapy or combined with amikacin, were the first-line empirical antibacterial treatment options for FN. Carbapenems were used as the first-line treatment for neutropenic fever in patients who were previously colonized or infected with extended-spectrum beta-lactamase (ESBL)-producing gram-negative bacilli or multidrug-resistant gram-negative bacilli (defined as lack of susceptibility to at least one agent in three or more antibiotic classes) [27], presented with septic shock (defined as sepsis with persistent hypotension requiring vasopressors to maintain a mean arterial pressure of ≥65 mm Hg and a serum lactate level of >2 mmol/L despite adequate volume resuscitation) [28] or with nosocomial pneumonia, received quinolone prophylaxis or broad-spectrum antibiotics within 10 days, or had a history of hospitalization in the intensive care unit for more than 72 h within the last 15 days. Vancomycin/teicoplanin was used when there was a suspicion or detection of septic shock, central-line-associated infection, previous colonization with methicillin-resistant *Staphylococcus aureus* (MRSA), or the detection of severe mucositis. Glycopeptides were stopped if blood cultures did not yield gram-positive bacteria and the patient was clinically stable. Colistin was reserved for patients who were already known to be colonized or previously infected with carbapenem-resistant gram-negative bacilli.

The appropriateness of the bacterial treatment was evaluated in patients with bacterial growth in their blood cultures. The initial empirical antimicrobial therapy was considered to be “appropriate” if the initial antibiotics that were administered after the blood cultures were collected included at least one antibiotic that was active in vitro and when the dosage and route of administration were in accordance with current medical standards, except in case of only being susceptible to aminoglycosides due to low serum levels. The antibacterial therapy was escalated (defined as broadening the spectrum of antibiotics) or de-escalated (defined as narrowing the spectrum of antibiotics) in blood-culture-positive patients according to antimicrobial susceptibility tests and clinical progress. Fever response to empirical antibiotics was defined as a patient being afebrile for at least three days after 48 h of empirical antibacterial treatment. Systemic antibiotics were stopped regardless of continuous fever or neutrophil count in clinically stable patients whose blood cultures were reported as negative.

During the study period, blood cultures were performed by using Bactec Plus Aerobic/F bottles (aerobic bottles) and Bactec Lytic/10 Anaerobic/F bottles (anaerobic bottles) incubated in the Bactec FX system. Species identification was carried out by using matrix-assisted laser desorption/ionization time-of-flight mass spectrometry (MALDI-TOF MS. Bruker, Bremen, Germany). Antimicrobial susceptibility tests were performed on a Phoenix system (Becton-Dickinson Diagnostic Systems, Sparks, MD, USA) and interpreted in accordance with the European Committee on Antimicrobial Susceptibility Testing criteria (https://www.eucast.org/clinical_breakpoints, accessed on 23 May 2021). All positive blood cultures with Gram-negative bacilli, *S. aureus*, or *Enterococcus* species were considered true bacteremia. Blood cultures with coagulase-negative staphylococci, diphtheroid, or viridans streptococci were considered true bacteremia if two blood cultures that were obtained in 24 h yielded the same bacteria.

### 4.4. Development of a Computerized Clinical Decision Support System

A web-based software application was developed. The application consisted of a user interface that ran in the browser and a backend hosted on a remote server. The HTTP protocol was used to communicate with the application server (link: https://fen.hemosoft.com/, accessed on 3 November 2021). The backend code used RESTful endpoints to establish URL-based communication with the server. In November 2021, the application data model was created using Hibernate ORM with a code-first approach.

The technical infrastructure of the FEN application consisted mainly of a decision-tree algorithm. A rule was defined as any separate branch of the decision tree that produced a result. The rules were created in collaboration with the domain expert (EY) and ID physicians (ZT, GM, OU, MA) according to the decision-tree algorithm, which was based on the institutional FN guidelines; these rules were predefined in the system and processed via the Builder software (version 1.0.0) pattern. The user could enter parameters and perform calculations to create rules.

**Figure 2:**
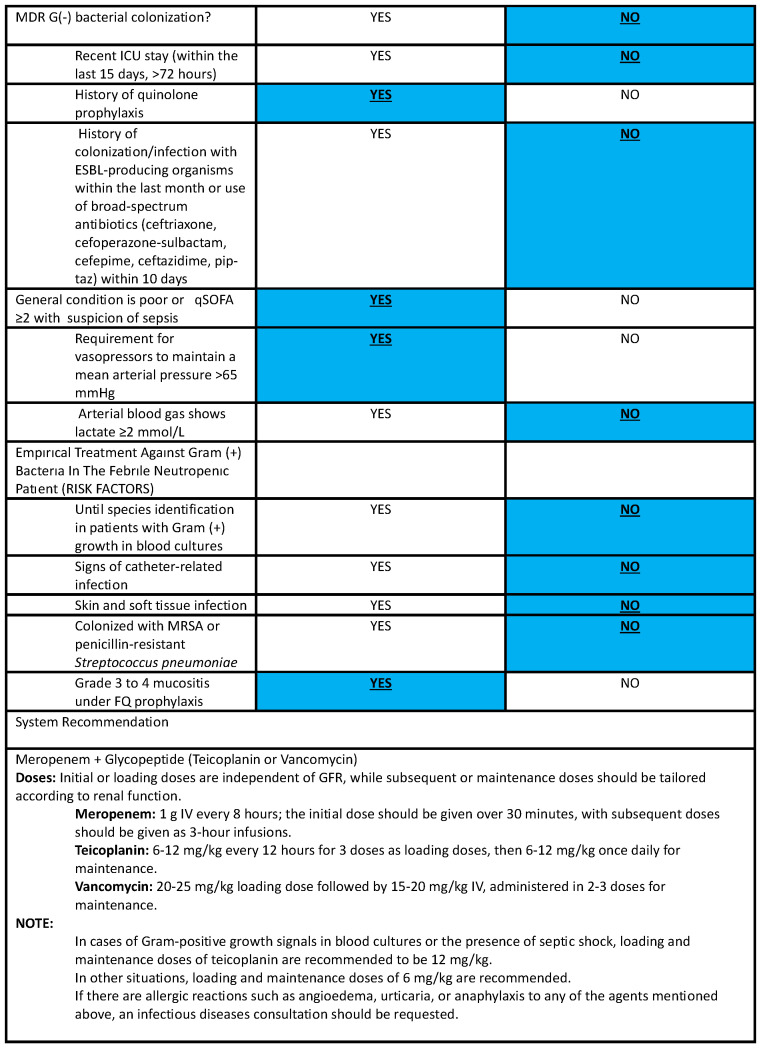
An empiric antibiotic treatment scenario. MDR (Multidrug–resistant), ICU (Intensive care unit), ESBL (Extended–spektrum beta–lactamases), Pip–taz (Piperacillin–tazobactam), qSOFA (quick Sequential Organ Failure Assessment), MRSA (Methicillin–resistant *Staphylococcus aureus*), GFR (Glomerular filtration rate).

Each separate branch of the decision tree that produces a result defines a rule. The software application allows the user to enter parameters and perform calculations to create rules. Inputs are mainly made by clicking on radio buttons and can vary from numeric to multiple-choice. In summary, the application uses all input parameters to fulfill the expected decision support for the user. Basically, the CDSS questions the predefined risk factors for each relevant scenario and then gives a recommendation according to the decision-tree algorithm. Figure 2 summarizes the CDSS’s performance in a sample scenario. As the CDSS is currently only accessible in Turkish and data entry is also confined to this language, a sample scenario was entered into the CDSS in Turkish. Subsequently, all steps and recommendations were translated into English while maintaining the overall format and appearance of the CDSS in English, Figure 2. An empiric antibiotic treatment scenario: A 59-year-old female diagnosed with acute lymphoblastic leukemia was admitted to the hematology ward for induction chemotherapy 13 days ago. Now, she is diagnosed with febrile neutropenia. The process included three questioning steps to recommend empirical antibacterial treatment. Questioning included the risk factors for multidrug-resistant gram-negative bacterial infection, the clinical condition, and the risk factors for multidrug-resistant gram-positive bacterial infection.

### 4.5. Evaluation of the Applicability of the CDSS

Four internal medicine and ID residents and three ID specialists were asked to utilize the CDSS in scenarios that closely resembled clinical encounters. They were informed via a verbal walkthrough, describing how to use the prototype CDSS application. In order to be certain whether the CDSS recommendations were consistent with the local guideline, the subjects were asked to use the existing intranet CDSS web portal on 50 (16 for antimicrobial prophylaxis, 13 for screening tests, 4 for diagnostic tests, 10 for empirical antibacterial treatment, and 7 for antifungal treatment) occasions and capture screenshots for each scenario. The outputs of these recordings were evaluated by two researchers (Z.T. and G.M.) in order to ascertain their compliance with the institutional protocol.

Once it was established that the CDSS recommendations were aligned with the institutional protocol, the descriptive data of febrile neutropenic patients with bacteremia were entered into the CDSS. The antibiotic recommendation provided by the CDSS was then compared with the antibiogram result.

### 4.6. Statistics

For statistical analysis, we divided the patients into two groups according to the presence (P1) or absence (P2) of the FN protocol at the time of diagnosis of the febrile neutropenic episode. Compliance with local guidelines was compared for each period. Continuous variables are presented as median/mean values and interquartile ranges/standard deviations, while categorical ones are presented as percentages. The chi-squared test or Fisher’s exact test was used for categorical variables, and Student’s *t*-test or the Mann–Whitney U-test was used for continuous variables according to a normality pattern. A *p*-value of <0.05 was accepted as significant in all statistical comparisons. Statistical analyses were performed by using IBM SPSS Statistics. version 22.0 (IBM Corp, Armonk, NY, USA).

The primary outcome measure was the achievement of a 20% increase in the rate of appropriate empirical treatment of FN in bacteremic patients. This specific threshold was chosen according to the results of a multicenter study including our hospital. Out of 431 febrile neutropenic patients with bacteremia, 67.8% received appropriate empirical treatment [29]. We aimed for a 20% increase in this rate. The statistical power for the outcome was calculated by using https://www.openepi.com/Power/PowerCohort.htm, last accessed on January 2024.

## 5. Conclusions

In conclusion, a local guideline in the local language with ongoing performance review and feedback to physicians may help provide a sustained improvement in the management of FN. The findings regarding the applicability of the CDSS are promising. Further studies are required to evaluate the role of CDSSs in the effective management of FN.

## Figures and Tables

**Table 1:** Distribution of the demographic and clinical characteristics of the patients according to the study periods.

	First Period (n = 91) (%)	Second Period (n = 136) (%)	All Patients (n = 227) (%)	*p*
Age (median, minimum–maximum) (years)	50 (18–82)	55 (18–82)	53 (18–82)	0.16
Sex. n (%)				0.71
Female	35 (38.5)	49 (36.0)	84 (37.0)	
Male	56 (61.5)	87 (64.0)	143 (63.0)	
Hematological malignancy	69 (75.8)	106 (77.9)	175 (7.1)	0.35
Acute myeloid leukemia	14 (20.3)	36 (34.0)	50 (28.6)	
Acute lymphocytic leukemia	11 (15.9)	17 (16.0)	28 (16)	
Multiple myeloma	8 (11.6)	19 (17.9)	27 (15.4)	
Non-Hodgkin lymphoma	30 (43.5)	27 (25.5)	57 (32.6)	
Hodgkin’s lymphoma	3 (4.3)	1 (0.9)	4 (2.3)	
Myelodysplastic syndrome	2 (2.9)	3 (2.8)	5 (2.9)	
Chronic lymphocytic leukemia	0 (0.0)	3 (2.8)	3 (1.7)	
Biphenotypic leukemia	1 (1.4)	0 (0.0)	1 (0.6)	
Solid tumors *	22 (24.2)	27 (19.9)	49 (21.6)	
None-malignant conditions **	0 (0.0)	3 (2.2)	3 (1.3)	
HSCT	22 (100.0)	44 (100.0)	66 (100.0)	0.21
Allogeneic HSCT	11 (50.0)	15 (34.1)	26 (39.4)	
Autologous HSCT	11 (100.0)	15 (100.0)	40 (60.6)	
Duration of neutropenia (median, minimum-maximum) days	6 (1–70)	7 (0–112)	7 (0–112)	0.01
Patients who did not recover from neutropenia	5 (5.5)	16 (11.8)	21 (9.3)	0.54

* Lung cancer (n = 17), breast cancer (n = 6), testicular cancer (n = 5), sarcoma (n = 4), pancreatic cancer (n = 4), prostate cancer (n = 2), esophageal cancer (n = 2), hepatoblastoma (n = 1), cholangiocarcinoma (n = 1), colon cancer (n = 1), glioblastoma multiforme (n = 1), gliosarcoma (n = 1), cervical cancer (n = 1), spinal cord tumor (n = 1), wilms tumor (n = 1), adenocarcinoma of unknown primary site (n = 1); ** Aplastic anemia (n = 1), myasthenia gravis (n = 1), and adrenoleukodystrophy (n = 1); all patients were recipients of hematopoietic stem cell transplantation (HSCT).

**Table 2:** Compliance with antibacterial and antifungal prophylaxis.

	Compliance Rate		
	First Period (P1)	Second Period (P2)	*p*
Antibacterial prophylaxis *			
Full compliance n (%)	11/33 (33.3)	25/61 (41.0)	0.53
Type of drug and dosage n, (%)	27/27 (100)	46/46 (100)	
Time of initiation and cessation n, (%)	11/33 (33.3)	25/61 (41.0)	0.53
Antifungal prophylaxis **			
Full compliance n, (%)	7/33 (21.2)	8/74 (10.8)	0.19
Type of antifungal drug n, (%)	29/33 (87.9)	65/71 (91.5)	0.72
Compliance with the dosage n, (%)	25/28 (89.3)	64/65 (98.5)	0.08
Time of initiation and cessation n, (%)	3/24 (23.1)	7/59 (17.5)	0.54

* Five patients were not included in the evaluation due to a lack of sufficient data or the development of febrile neutropenia under systemic antibacterial treatment. ** A total of 22 patients were excluded from the evaluation since they were under systemic antifungal treatment or there were insufficient data when febrile neutropenia was detected.

**Table 3:** A comparison of risk factors for antibacterial resistance, indications for empirical glycopeptide treatment, and epidemiological characteristics of bloodstream infections.

	First Period (P1) (n = 91)%	Second Period (P2) (n = 136)%	All Patients (n = 227)%	*p*
Duration of neutropenia (median, minimum–maximum) days	6 (1–70)	7 (0–112)	7 (0–112)	0.01
Patients who did not recover from neutropenia	5 (5.5)	16 (11.8)	21 (9.3)	0.54
Risk factors for antibacterial resistance				
Levofloxacin prophylaxis	39 (42.9)	71 (52.2)	110 (48.5)	0.18
Broad spectrum antibiotic consumption in the last month	63 (69.2)	98 (72.1)	161(70.9)	0.64
Septic shock	5 (5.5)	10 (7.4)	15 (6.6)	0.58
Previous colonization with a multidrug resistant bacterium	3 (3.3)	1 (0.7)	4 (1.8)	0.3
Presence with hospital acquired pneumonia	5 (5.5)	4 (2.9)	9 (4)	0.49
Receipt of care in the intensive care unit >72 h in the last 6 months	4 (4.4)	2 (1.5)	6 (2.6)	0.22
Indications for empirical glycopeptide treatment				
Grade 3 or 4 mucositis	4 (4.4)	8 (5.9)	12 (5.3)	0.76
Cellulitis	2 (2.2)	9 (6.6)	11 (4.8)	0.20
Previous Methicillin-resistant *Staphylococcus aureus* colonization	0 (0.0)	0 (0.0)	0 (0.0)	-
Pain around central venous line	0 (0.0)	1 (0.7)	1 (0.4)	1.0
Erythema around central venous line	0 (0.0)	5 (3.7)	5 (2.2)	0.09
Perianal pain	3 (3.3)	6 (4.4)	9 (4)	0.75
Number of patients with bacteremia	24 (25)	42 (30.9)	66 (29.1)	0.46
Bacteremia with a multidrug resistant Gram-negative bacilli	10 (10.9)	12 (8.8)	22 (9.7)	0.59
3rd-generation cephalosporin-resistant Enterobacterales	8	11		
3rd-generation cephalosporin-resistant non-fermentating gram-negative bacilli	1	0		
Carbapenem-resistant Gram-negative bacilli	1	1		
Bacteremia with a multidrug-resistant gram-positive bacteria	5 (5.5)	3 (2.2)	8 (3.5)	0.21
Methicillin-resistant Staphylococci	4	2	6	
Ampicillin-resistant *Enterococcus faecium*	0	0	1	
Penicillin-resistant *Streptococcus viridans*	1	1	1	

**Table 4:** Antimicrobial treatment characteristics of the patients.

	First Period (n = 91)(%)	Second Period (n = 136)(%)	All Patients (n = 227)(%)	*p* Value
Empirical antibacterial treatment compliance with the local guideline	72 (79.1)	118 (86.8)	190 (83.7)	0.13
Appropriate empirical antibacterial treatment in patients with positive blood cultures (n = 66)	14/24 (58.3%)	37/42 (88.1%)	51/66 (77.3%)	0.006
Escalation of empirical antibacterial treatment	45 (49.5)	48 (35.3)	93 (41.0)	0.03
Reasons for escalation of empirical antibacterial treatment				0.35
Persistent fever	22 (48.9)	27 (56.3)	49 (52.7)	
Clinical deterioration	11 (12.1)	14 (29.2)	25 (26.9)	
Bacteremia by a resistant bacterium	12 (26.7)	7 (14.6)	19 (20.4)	
Duration between the day of empirical antibacterial treatment and escalation (Median, minimum-maximum) days	3 (1–9)	4 (1–13)	4 (1–13)	0.03
De-escalation of empirical antibacterial treatment	12 (13.2)	22 (16.2)	34 (15)	0.54
Defervence with first line antibacterial treatment	32 (35.2)	79 (58.1)	111 (48.9)	0.001
Duration of antibacterial treatment ≤ 7 days	20 (21.9)	47 (34.5)	67 (29.5)	0.02
Number of patients who received empirical antifungal treatment due to persistent fever	8 (8.8)	19 (14)	27 (11.8)	0.24
Number of patients who received antifungal treatment with a diagnosis of invasive fungal disease	6 (6.6)	12 (8.8)	18 (7.99)	0.54
Invasive aspergillosis *	4	9	13	
Fungemia	0	2	2	
Candida esophagitis	1	0	1	
Fungal sinusitis	1	2	3	
30-day mortality	16 (17.6)	22 (16.2)	38 (16.7)	0.78

* Invasive aspergillosis was diagnosed according to the European Organization for Research and Treatment of Cancer (EORTC) and the National Institute of Allergy and Infectious Diseases Mycoses Study Group (MSG) criteria [20].

## Data Availability

The data that support the findings of this study are available from the corresponding author (Z.T.) upon reasonable request.

## Appendix A

Power calculation for the comparison of appropriate antibacterial treatment in patients with bacteremia between two periods (https://www.openepi.com/Power/PowerCC.htm, last accessed on January 2024).

**Table A1:** Power calculation for the comparison of appropriate antibacterial treatment in pa-tients with bacteremia between two periods.

Power for Unmatched Case–Control Studies	
	Input Data
Two-sided confidence interval (%)	95
Number of cases	91
Percent of exposure among cases (%)	58
Number of controls	136
Percent of exposure among controls (%)	88
Odds Ratio	0.19
Power based on:	
Normal approximation	99.93%
Normal approximation with continuity correction	99.87%

Results from OpenEpi, Version 3, open-source calculator—PowerCC. Print from the browser with ctrl-P or select text to copy and paste to other programs.
